# Investigating the hemiretinal asymmetry in emotion processing as a function of spatial frequency

**DOI:** 10.1098/rspb.2024.1909

**Published:** 2024-11-06

**Authors:** Eleanor Moses, Zhou Yu, Jessica Taubert, Alan J. Pegna

**Affiliations:** ^1^ School of Psychology, The University of Queensland, Brisbane, Queensland 4072, Australia

**Keywords:** EEG, hemiretina, spatial frequency, subcortical, fearful faces

## Abstract

The subcortical visual pathway to the amygdala has long been considered a rapid and crude stream for processing emotionally salient information that is reliant on low spatial frequency (LSF) information. Recently, research has called this LSF dependency into question. To resolve this debate, we take advantage of an anatomical hemiretinal asymmetry, whereby the nasal hemiretina sends a higher proportion of information through the subcortical pathway than the temporal hemiretina. We recorded brain activity using electroencephalography (EEG) in human participants (*N* = 40) while they completed a monocular viewing paradigm. Pairs of faces (one fearful and one neutral, or both neutral) were projected simultaneously to the nasal and temporal hemiretina in three contrast-equated blocks; faces filtered to display only (i) LSF, (ii) high spatial frequency (HSF), or (iii) unfiltered information (broadband spatial frequency; BSF). BSF fearful faces were found to produce a greater naso-temporal asymmetry, with greater N170 amplitudes evoked by BSF faces in the nasal field, compared to HSF faces. Conversely, the naso-temporal asymmetry for LSF fearful faces did not differ between BSF and HSF. Collectively, these findings provide crucial evidence that the subcortical pathway carries combined spatial frequency visual signals, with a potential bias against HSF content.

## 1. Introduction

Visual signals are primarily projected from the retina through the geniculostriate pathway, to the lateral geniculate nucleus (LGN) and then the primary visual cortex (V1). However, there is also a parallel subcortical pathway, which carries visual signals from the retina to the superior colliculus [1]. From the superior colliculus, the subcortical pathway projects to the pulvinar and the amygdala [2,3]. It has been suggested that this pathway, which is phylogenetically older than the cortical one, allows for the rapid detection of threat-related visual stimuli, such as snakes, spiders and fearful emotional expressions, which all act as cues for imminent threats to survival [2]. However, this pathway’s functional importance has been difficult to establish because of its deep anatomical location and consequently the complexity of determining its temporal dynamics. Here, our goal was to take advantage of a known anatomical asymmetry to extricate the responses of this subcortical pathway and determine whether it is biased towards low spatial frequency (LSF) information, thereby indicating its reliance on more crude visual information.

The superior colliculus consists primarily of magnocellular neurons receptive to LSF information over small temporal increments [4]. Therefore, information processed in the subcortical visual pathway is assumed to be ‘coarse’ in that it is reliant on low-resolution visual signals. Confirming this idea in the emotional domain, behavioural performance [5,6], blood oxygen level-dependant (BOLD) subcortical activation [7–9] and early electrophysiological correlates of emotional faces were all found to be enhanced for LSF compared to high spatial frequency (HSF) components of the stimuli [10,11]. Accordingly, reliance on LSF stimuli is thought by some to be a key physiological property of the subcortical visual pathway, allowing faster responses to visual stimuli based on crude visual information [12–17]. However, this assertion lacks conclusive support.

Neuroimaging studies examining the responses to LSF stimuli in the subcortical pathway have yielded mixed results [18]. For example, functional magnetic resonance imaging (fMRI) has shown that both HSF faces and LSF faces drive activity in the amygdala [9,19]. However, these results are constrained by the temporal resolution of fMRI, which does not allow temporally distinct activations to be identified and thus may be insufficient to establish the contribution of the faster subcortical pathway. Interestingly, a more recent study using magnetoencephalography, which provides a high temporal resolution, employed dynamic causal modelling and demonstrated that the subcortical pathway was insensitive to spatial frequency content [20]. In light of these findings, these authors argued that the subcortical pathway is not differentially sensitive to spatial frequency (SF) manipulations but rather to changes in the contrast envelope [20]. Therefore, it remains unclear whether the subcortical pathway relies on LSF content to rapidly detect threat-related stimuli, such as fearful faces, in the visual environment.

While neuroimaging studies hint at an interaction between fearful faces and SF content, they cannot attribute these differences to subcortical involvement directly since brain function in healthy participants recruits all visual pathways' stimuli in parallel. To tease apart the relative contributions of the subcortical and cortical routes, we here relied on differences in projections between the nasal and temporal retinae.

**Figure 1.**
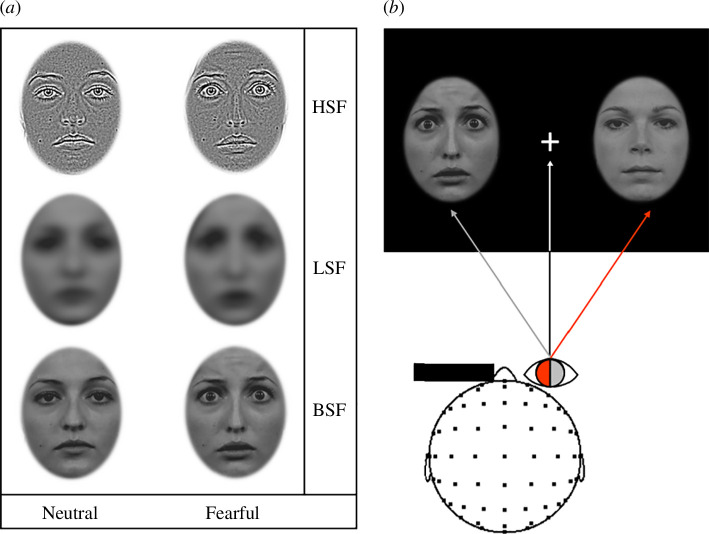
(*a*) Examples of the fearful and neutral stimuli used in the experiment. All stimuli were filtered to contain HSF or LSF information or were left unfiltered (BSF). (*b*) A diagram illustrating the nasal and temporal hemiretina presentation (nasal in red and temporal in grey). The left eye is occluded and the participant fixates on the central cross. The left stimulus (here a fearful face) is therefore in the nasal viewing field and is processed solely by the temporal hemiretina (grey shading and arrow), and the right stimulus (neutral face) is in the temporal viewing field and is processed solely by the nasal hemiretina (red shading and arrow).

For each eye, the nasal hemiretina receives information from the temporal visual field, while the temporal (lateral) hemiretina receives information from the nasal visual fields (figure 1). Each hemiretina projects equally to the geniculostriate route; however, there is a key naso-temporal asymmetry in the proportion of projections to the subcortical route that can be exploited to isolate the tuning properties of subcortical visual pathway. Indeed, detailed lesion and structural studies in non-human primate models have demonstrated that the superior colliculus receives a greater number of projections from the nasal hemiretina than the temporal hemiretina [3,21,22]. Thus, while binocular viewing of visual stimuli will stimulate both nasal and temporal hemiretinae, monocular viewing of lateralized stimuli will stimulate the temporal hemiretina if only the ipsilateral eye is used and the nasal hemiretina if only the contralateral eye is used. Hence, monocular viewing can allow the nasal or temporal hemiretina to be stimulated in isolation, leading, respectively, to greater or lesser activation of the subcortical route. This approach has been confirmed in several investigations. For example, in an fMRI study, visual stimulation of the nasal hemiretina drove more activity in the superior colliculus than presentations to the temporal hemiretina, while no differences were found in the retinogeniculate pathway or V1 [23]. Neuropsychological studies have shown the presence of a blindsight phenomenon for visual stimuli presented to the nasal, but not temporal hemiretina [24,25]. Finally, behavioural studies of neurotypical adults have reported faster detection [26], greater attentional capture [27–29] and greater capacity to obstruct attention [30] when visual stimuli are presented to the nasal hemiretina compared to temporal hemiretina.

In sum, there is a confluence of evidence indicating that the superior colliculus receives more input from the nasal hemiretina than the temporal hemiretina. Based on these observations, here we asked whether neural responses to fearful faces presented to the nasal hemiretina—and thus the subcortical pathway—were modulated by spatial frequency content.

In past electrophysiological research, face stimuli presented to the nasal hemiretina have evoked a greater N170 response (a known neural marker of face processing) than face stimuli presented to the temporal hemiretina [31–33]. The same hemiretinal asymmetry is thought to be the source of an asymmetry in attentional deployment, with nasal hemiretina presentations evoking a greater N2pc (a known neural marker of attentional capture) than temporal hemiretina presentations [34]. Another neural marker of note is the early posterior negativity (EPN), which is thought to reflect the encoding of affective information and the degree of allotted visual attention [35–40]. The EPN has not previously been assessed via the hemiretinal asymmetry and could provide unique insight into the interaction between SF and emotion on related encoding and attentive processes. Building on these previous observations, in the current study we employed a monocular viewing paradigm in an event-related potential (ERP) experiment to test the sensitivity of the subcortical pathway to spatial frequency content in fearful faces while crucially correcting for contrast between SF stimuli conditions. If the subcortical pathway is reliant on LSF information [7,8], we would expect to see a disproportional neural response to LSF fearful faces that is dependent on the hemiretina condition (nasal > temporal). Meanwhile, HSF faces will evoke the same neural responses in both hemiretina conditions (nasal = temporal). Alternatively, if the subcortical pathway is not reliant on LSF information [20], we would expect to see similar neural responses to LSF and HSF faces, regardless of the hemiretina condition.

## 2. Methods

### (a) Participants

Participants were volunteers and undergraduate students who took part in the experiment in exchange for course credit. Data were collected for 40 participants, with four being excluded in the pre-processing stage owing to noisy data. This left a final sample of 36 participants (10 male; age *M* = 24.90, s.d. = 6.15 years). The study was conducted with the approval of the University of Queensland Institutional Human Research Ethics Committee (2022/HE000106).

### (b) Apparatus

All stimuli were presented on a 24-inch ASUS LCD monitor model VG248QE (resolution: 1920 × 1080 pixels; refresh rate: 60 Hz). Responses were recorded on a Dell KB522p keyboard. Participants viewed stimuli from a distance of 50 cm, and the experiment was coded using PsychoPy v.2022.1.1 [41].

### (c) Scales and stimuli

Faces were selected from the Radboud Faces Database [42] and 10 identities were selected each for males and females. Two expressions were used for each identity (neutral and fearful). Stimuli were initially cropped to 512 × 512 squares, then a MATLAB [43] script developed by Perfetto *et al.* [44] was used to filter the stimuli.

Each face was prepared for three conditions: unfiltered, LSF and HSF. Thus, there were 120 stimuli in total (20 identities displaying two expressions, in three filtering conditions). Faces in the unfiltered condition (broadband spatial frequency; BSF) were not filtered, faces in the LSF condition were filtered to display only LSF information with a cut-off of <8 cpi (approx. 0.85 cpd) and faces in the HSF condition were filtered to display only HSF information using a cut-off of >32 cpi (cycles per image; approx. 3.4 cpd or cycles per degree), both using a Butterworth2 filter [44], in line with suggestions by Jeantet *et al*. [10]. Critically, all filtered stimuli were subsequentially processed to equate the contrast values across the LSF and HSF conditions using the SHINE toolbox [45]. Each face stimulus was then imported to Gimp v.2.0 [46] for elliptical cropping. Faces were cropped to a vertical ellipse of 337 × 256.12 pixels such that only the facial expression was visible (and not facial contours). The remaining area was set to be transparent (see figure 1 for example stimuli).

Faces were presented against a black background (red green blue (RGB): 0, 0, 0). A fixation cross was presented at the centre of the screen (font = Open Sans, height/width = 0.40°; 26.45 pix, colour = white; RGB: 255, 255, 255, or grey; RGB: 148, 148, 148). Faces were presented bilaterally 7° to the left or right of the fixation cross, as in previous monocular hemiretinal paradigms [32,34]. Each face subtended 10.20° (337 pix) in height and 7.62° (256.12 pix) in width. Bilateral face presentations were used to ensure the emergence of an N2pc, as well as to minimize saccadic movements and attention shifts.

To ensure that the sample was neurotypical, a standard battery of questionnaires was administered. These included the State-Trait Anxiety Inventory (STAI; [47]), Autism Quotient (AQ; [48]), Liebowitz Social Anxiety Scale (LSAS; [49]) and the Beck Depression Index (BDI; [50]).

### (d) Design and procedure

The experiment used a 2 (viewing eye: left, right) × 2 (hemiretina presentation: nasal, temporal) × 2 (stimuli: neutral/neutral or neutral/fearful face) × 3 (spatial frequency: LSF, HSF, BSF) mixed design. Viewing eye was counterbalanced across subjects and all other variables were within-subjects.

Before the experimental task, participants completed the STAI, AQ, LSAS and BDI.

Participants had their left or right eye covered using an eyepatch and were informed of the instructions both verbally and on-screen. Participants were told to fixate on the centre of the screen on the fixation cross and to ignore the faces that would appear bilaterally.

**Figure 2.**
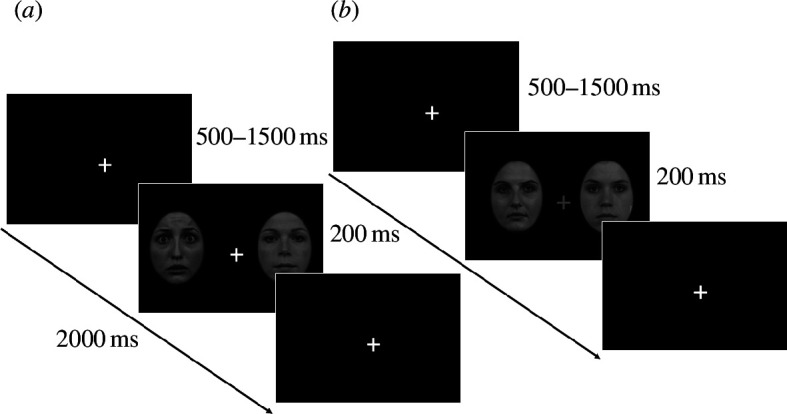
Examples of the experimental trials. (*a*) An unfiltered fear-left trial with no fixation cross-change. (*b*) An unfiltered neutral trial with the target fixation cross-change (to be responded to with a space-key press).

Each trial took 2 s and began with a central fixation screen. Two faces were then presented bilaterally for 200 ms, at a varied onset between 0.5 and 1.0 s from the trial start. These two faces were either neutral/neutral (neutral trials), neutral left and fearful right (fearful-right trials) or neutral right and fearful left (fearful-left trials; see figure 1). For participants with a left viewing eye (right covered), left fearful stimuli were viewed by the nasal hemiretina and right fearful stimuli were viewed by the temporal hemiretina. The opposite was true for participants with a right viewing eye (left covered; see figure 1). In 9% of the trials, the fixation cross would change from white to grey for 200 ms at a varied onset between 0 and 1.5 s from the trial start (see figure 2). Participants were asked to maintain their gaze on the fixation cross and press the space key when they observed a colour change in the cross. Stimuli were presented in three blocks so that each block showed either LSF, HSF or BSF faces. The order of these blocks was counterbalanced across participants. Each block consisted of 330 total trials (110 for each of the face presentation variations: neutral, fear left, fear right). After the removal of the 10% of fixation colour change trials, 100 usable trials per condition remained, with 300 trials per block, and 900 trials total. Each block had a self-paced break halfway through and at the end of the block. Blocks lasted 10 minutes on average, and thus the total testing time was approximately 30 minutes.

### (e) EEG recording and analysis

Continuous EEG was recorded at 1024 Hz using an AD-Box BioSemi ActiveTwo amplifier (BioSemi, Amsterdam, the Netherlands), with 64 electrodes placed in accordance with the international 10–20 system, a common mode sense active and driven right leg passive electrode serving as reference and ground, and two external electrodes placed vertically below (EXG2) and at the lateral canthus of the viewing eye (EXG1), respectively, to measure eye movements.

#### (i) EEG pre-processing

EEG data were pre-processed using BrainVision Analyzer v. 2.2.0 (Brain Products GmbH, Gilching, Germany; see https://ils-labs.wp.hum.uu.nl/resource/brainvision-analyzer-2-2-0/). Data were downsampled from 1024 to 512 Hz. Next, any noisy electrodes were replaced with topographic interpolation (a cut-off was used such that any participants with over four interpolated electrodes would be removed from analysis; none were removed). Data were re-referenced to the average of all 64 electrodes (excluding facial electrodes). Data were filtered with a high-pass filter of 0.1 Hz, a low-pass filter of 40 Hz and a notch filter at 50 Hz. An eye-movement channel was created from the difference waveform of EX1 and EX2. An independent component analysis was conducted to remove artefacts in the data owing to eyeblinks. Data were segmented around a marker time-locked to the presentation of stimuli, with a −100 ms baseline before stimuli presentation and 600 ms after. Next, semi-automatic artefact rejection was conducted to remove trials where eye movements occurred (where the eye movement channel exceeded ±60 µV). Semi-automatic artefact rejection was then conducted to remove trials where muscle movements occurred (any trial where any of the 64 electrodes exceeded ±100 µV). Average ERPs for each condition (nasal and temporal presentations of fearful faces and neutral–neutral faces, for BSF/LSF/HSF) were created for each participant. Data from three parietal electrodes on each hemisphere were then pooled together to create a left (PO7, P7, P9) and right (PO8, P8, P10) parietal response to be used for analysis. These electrodes were selected based on previous hemiretinal EEG research [34].

#### (ii) ERP analysis

#### Emotion × SF ERP analysis

**Figure 3.**
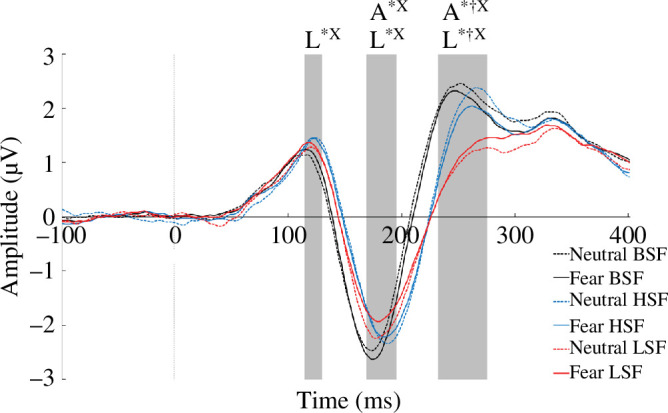
ERPs for trials with two neutral faces (dotted) and trials with one neutral and one fearful face (solid). Fearful face ERPs are collapsed here over presentation field (nasal or temporal hemiretina). Each line represents the response over bilateral parietal electrodes (average of PO7, P7, P9, PO8, P8, P10). Evoked responses are shown for BSF (black), HSF (blue) and LSF (red) faces. Note, L indicates significant differences for latency, A indicates significant differences for amplitude, * indicates significant main effect of spatial frequency, ^†^ indicates significant main effect of emotion, ^X^ indicates a significant interaction between emotion and spatial frequency. Grey boxes indicate general epochs of activation, which were detected through peak detection for each participant.

An initial comparison of fearful faces (regardless of hemiretinal presentation) with neutral faces by SF was conducted to determine whether emotion-based differences interacted with SF. For each participant, left- and right-pooled electrodes were averaged to create a measure of both parietal sets. ERPs to fearful faces were averaged across left and right viewing field presentations to create one ‘fearful’ condition, and this was compared with trials where two neutral faces were presented (see figure 3). Early components (P1, N170 and EPN) were examined. The latency and amplitude were determined and exported for each participant and each condition through semi-automatic peak detection, where the first global maximum (P1), first global minimum (N170) and second global maximum (EPN) were determined. Amplitude was exported as the average of the amplitude ±5 ms for P1 and N170 around the detected peak and ±15 ms for EPN around the detected peak. Analyses were 2 (emotion: fearful, neutral) × 3 (SF: BSF, HSF, LSF) repeated measure ANOVAs and all *post hoc* comparisons were Bonferroni corrected.

#### HemiRetina × SF ERP analysis

**Figure 4.**
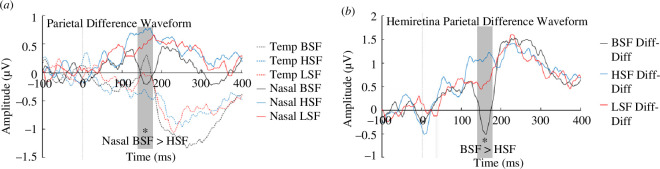
Difference waveforms in response to fearful faces presented to the nasal and temporal hemiretina. (*a*) The ‘N2pc’ difference waveform calculated as the difference between electrodes contralateral and ipsilateral to the field of stimuli presentation. Lines represent difference waveforms for BSF, LSF and HSF faces presented to the temporal or nasal hemiretina. (*b*) The difference waveform between temporal and nasal hemiretina presentations; calculated as the difference between the N2pc scores (*a*) for nasal and temporal hemiretina presentations. Lines represent difference of difference waveforms for BSF, LSF and HSF.

The analysis described in the above section focused on the relationship between SF and emotion, regardless of facial expressions or visual field. In contrast, the hemiretinal analysis focuses specifically on fearful face presentations of varying SFs. Hemiretinal asymmetry analysis requires the comparison of stimuli presented solely to an isolated hemiretina. Neutral faces were either presented to both hemiretinae simultaneously or alongside fearful faces. Thus, the asymmetry comparison could only be conducted for fearful faces. To determine whether the asymmetry for fearful face processing between nasal and temporal hemiretina differed as a function of spatial frequency, we examined difference waveforms. First, a classic ‘N2pc’ difference waveform was calculated. For each participant, this was calculated from contralateral—ipsilateral electrodes (with reference to the visual field of presentation; figure 4*a*). For participants with a left viewing eye, temporal presentations (right visual field) were left parietal—right parietal electrodes, and nasal presentations (left visual field) were right parietal—left parietal electrodes, for a right viewing eye, this was the inverse (temporal was left visual field, thus calculated as right parietal—left, and nasal was right visual field, thus left parietal—right). Thus, we computed two N2pc difference waveforms, one for temporal and one for nasal retinal presentations irrespective of viewing eye or side of presentation. Next, to determine if the asymmetry in responses to each hemiretina differed with spatial frequency, we calculated the difference between these difference waveforms for each participant. We subtracted the temporal retinal presentation N2pc difference waveform from the nasal retinal presentation N2pc difference waveform and calculated this separately for BSF, LSF and HSF faces (figure 4*b*). This produced a measure of the asymmetry between hemiretinal presentations for each SF condition. The ultimate goal being to determine whether difference of differences waveforms for LSF and HSF differed significantly from the waveform for BSF faces (where the typical asymmetry would occur). Henceforth these difference of differences waveforms will be referred to as hemiretinal difference waveforms.

Analyses were conducted on peaks P1, N1 and EPN, and epochs were determined by visual analysis of the grand averages of these hemiretinal difference waveforms. For each participant, the values for epochs from 75 to 100 (P1.1), 115–125 (P1.2), 155–170 (N170) and 205–245 (EPN) were exported for analysis. One-way repeated measures ANOVAs were conducted for amplitude at each of the three peaks for each spatial frequency (BSF, LSF and HSF). Bonferroni corrections were applied to all *post hoc* comparisons. If any significant differences in asymmetry were detected, these were followed up with comparisons of hemiretina to determine the origin of any difference using a 2 (hemiretina: temporal, nasal) × 3 (spatial frequency: BSF, HSF, LSF) repeated measures ANOVA.

## 3. Results

Data can be accessed at https://osf.io/83ejv/.

### (a) Emotion × SF ERP

#### (i) The P1 event potential

**Amplitude**. There was no main effect of spatial frequency (*p* = 0.660) or emotion (*p* = 0.668) on P1 amplitude and the interaction was not significant (*p* = 0.943).

**Latency**. There was a main effect of spatial frequency on P1 latency, *F* (2, 35) = 5.43, *p* = 0.006, $\eta_{p}^{2}$ = 0.13. *Post hoc* comparisons revealed that P1 latencies to both BSF faces (*M* = 111.11, s.d. = 18.29; *t* = −2.65, *p*_bonf_ = 0.036, Cohen’s *d* = −0.31) and LSF faces (*M* = 111.33, s.d. = 18.47; *t* = −2.81, *p*_bonf_ = 0.023, Cohen’s *d* = 0.30) were earlier than to HSF faces (*M* = 116.89, s.d. = 19.09). There was no effect of emotion (*p* = 0.872). There was a significant interaction between the two, *F* (2, 35) = 4.65, *p* = 0.013, *$\eta_{p}^{2}$* = 0.12. *Post hoc* comparisons revealed significantly later latencies to neutral HSF than to neutral BSF faces (*M* = 119.52, s.d. = 18.15; *M* = 109.27, s.d. = 19.50, respectively; *t* = −4.12, *p*_bonf_ = 0.001, Cohen’s *d* = −0.55) and to neutral HSF than to neutral LSF faces (*M* = 110.95, s.d. = 19.25; *t* = 3.44, *p*_bonf_ = 0.012, Cohen’s *d* = 0.46). No other comparisons were significant (*p*_bonf_ < 0.05).

#### (ii) The N170 event potential

**Amplitude**. There was a main effect of spatial frequency on N170 amplitude, *F* (2, 70) = 6.87, *p* = 0.002, *$\eta_{p}^{2}$* = 0.16. *Post hoc* comparisons revealed significant differences between BSF (*M* = −3.66, s.d. = 2.92) and LSF faces (*M* = −2.95, s.d. = 2.58; *t* = −3.20, *p*_bonf_ = 0.011, Cohen’s *d* = −0.24), but no significant differences with HSF faces (*M* = −3.14, s.d. = 2.55; *p* > 0.05). There was no effect of emotion (*p* = 0.868). There was a significant interaction *F* (2, 70) = 3.32, *p* = 0.042, $\eta_{p}^{2}$ = 0.08. *Post hoc* comparisons revealed a significantly larger peak evoked to fearful BSF (*M* = −3.60, s.d. = 3.03) than fearful LSF faces (*M* = −2.75, s.d. = 2.61; *t* = −3.39, *p*_bonf_ = 0.015, Cohen’s *d* = −0.25) and neutral LSF faces (*M* = −2.91, s.d. = 2.57; *t* = 3.32, *p*_bonf_ = 0.019, Cohen’s *d* = 0.24), and a larger peak to neutral BSF (*M* = −3.12, s.d. = 2.90) than fearful LSF faces (*M* = −2.75, s.d. = 2.61; *t* = −4.41, *p*_bonf_ < 0.001, Cohen’s *d* = −0.31); no other comparisons were significant (*p*_bonf_ > 0.05).

**Latency**. There was a main effect of spatial frequency, *F* (2, 70) = 5.43, *p* = 0.006, *$\eta_{p}^{2}$*= 0.13. *Post hoc* comparisons revealed that all SFs were significantly different. N170 peaks for both BSF (*M* = 168.08, s.d. = 15.86; *t* = −2.65, *p*_bonf_ = 0.036, Cohen’s *d* = −0.31) and LSF faces (*M* = 178.15, s.d. = 18.49; *t* = 2.81, *p*_bonf_ = 0.023, Cohen’s *d* = 0.29) were earlier than HSF faces (*M* = 181.85, s.d. = 15.27). There was no effect of emotion, *F* (1, 70) = 0.03, *p* = 0.872. There was an interaction [2,51] = 4.65, *p* = 0.013, $\eta_{p}^{2}$ = 0.12. *Post hoc* comparisons found HSF neutral faces (*M* = 119.52, s.d. = 18.15) evoked slower peaks than both LSF (*M* = 110.95, s.d. = 19.25; *t* = 3.45, *p*_bonf_ = 0.012, Cohen’s *d* = −0.46) and BSF neutral faces(*M* = 109.27, s.d. = 19.50; *t* = −4.12, *p*_bonf_ = 0.001, Cohen’s *d* = −0.55).

#### (iii) The EPN deflection

**Amplitude**. There was a main effect of spatial frequency, *F* (2, 35) = 13.52, *p* < 0.001, $\eta_{p}^{2}$ = 0.28. *Post hoc* comparisons revealed that LSF faces (*M* = 1.86, s.d. = 1.78) evoked a more negative amplitude than both BSF (*M* = 3.04, s.d. = 2.20; *t* = 5.62, *p*_bonf_ < 0.001, Cohen’s *d* = 0.60Z) and HSF faces (*M* = 2.67, s.d. = 1.85; *t* = 4.19, *p*_bonf_ < 0.001, Cohen’s *d* = 0.41); BSF and HSF faces were not different (*p*_bonf_ > 0.05). There was a main effect of emotion, *F* (1, 35) = 9.55, *p* = 0.004, $\eta_{p}^{2}$ = 0.21, such that neutral faces (*M* = 2.64, s.d. = 2.06) evoked lesser negative deflections than fearful faces (*M* = 2.40, s.d. = 2.01). There was a significant interaction *F* (2, 35) = 3.17, *p* = 0.048, $\eta_{p}^{2}$ = 0.083. *Post hoc* comparisons revealed that both LSF fearful (*M* = 1.87, s.d. = 1.76) and LSF neutral faces (*M* = 1.87, s.d. = 1.76) evoked a greater negative deflection than all other faces except for HSF fearful (BSF fearful: *M* = 2.91, s.d. = 2.22; *t*_(LSF fearful)_ = 4.30, *p*_bonf_ < 0.001, Cohen’s *d* = 0.54; *t*_(LSF neutral)_ = −4.18, *p*_bonf_ < 0.001, Cohen’s *d* = −0.53; BSF neutral: *M* = 3.17, s.d. = 2.25; *t*_(LSF fearful)_ = 5.28, *p*_bonf_ < 0.001, Cohen’s *d* = 0.67; *t*_(LSF neutral)_ = 5.27, *p*_bonf_ < 0.001, Cohen’s *d* = 0.66; and HSF neutral: *M* = 2.89, s.d. = 1.90; *t*_(LSF fearful)_ = 4.16, *p*_bonf_ = 0.001, Cohen’s *d* = 0.53; *t*_(LSF neutral)_ = 4.13, *p*_bonf_ = 0.001, Cohen’s *d* = 0.52). HSF fearful faces (*M* = 1.52, s.d. = 1.82) evoked significantly more negative deflections than HSF neutral faces (*t* = 3.55, *p*_bonf_ = 0.009, Cohen’s *d* = 0.22). No other comparisons were significant.

**Latency**. There was a main effect of spatial frequency, *F* (2, 35) = 16.36, *p* < 0.001, $\eta_{p}^{2}$ = 0.32, and *post hoc* comparisons revealed that BSF faces (*M* = 244.19, s.d. = 25.38) evoked earlier peaks than HSF (*M* = 256.96, s.d. = 17.58; *t* = −4.76, *p*_bonf_ < 0.001, Cohen’s *d* = −0.57) and LSF faces (*M* = 260.48, s.d. = 23.02; *t* = −4.43, *p*_bonf_ < 0.001, Cohen’s *d* = −0.73). There was a main effect of emotion, *F* (1, 35) = 10.35, *p* = 0.003, Cohen’s *d* = 0.23, such that fearful faces (*M* = 250.47, s.d. = 19.88) evoked earlier peaks than neutral faces (*M* = 257.29, s.d. = 19.88). The interaction was not significant, *p* > 0.05.

### (b) HemiRetina × SF ERP

#### (i) The P1 event potential in the time window: 75–100 ms

There was no significant difference between any SF for P1 amplitude.

#### (ii) The P1 event potential in the time window: 115–125 ms

There was no significant difference between any SF for P1 amplitude.

#### (iii) The N170 event potential in the time window: 155–170 ms

There was a significant main effect of spatial frequency, *F* (2, 70) = 4.41, *p* = 0.016, $\eta_{p}^{2}$ = 0.112. *Post hoc* tests revealed that the differences between contralateral and ipsilateral electrodes for temporal and nasal hemiretina presentations were significantly different for BSF (*M* = −0.296, s.d. = 4.07) compared to HSF faces (*M* = 1.17, s.d. = 4.71; *t* = −3.20, *p*_bonf_ = 0.014, Cohen’s *d* = −0.34). There were no other significant comparisons, *p* > 0.05. A follow-up comparison investigating the origin of this difference revealed no main effects but a significant interaction, *F* (2, 70) = 4.41, *p* = 0.016, $\eta_{p}^{2}$ = 0.11. *Post hoc* comparisons revealed a significant difference between contralateral–ipsilateral difference waveforms evoked in response to BSF faces presented to the nasal hemiretina (*M* = −0.16, s.d. = 2.07) and HSF faces presented to the nasal hemiretina (*M* = 0.75, s.d. = 2.45), *t* = −3.17, *p*_bonf_ = 0.029, Cohen’s *d* = −0.40.

#### (iv) The EPN event potential in the time window: 205–245 ms

There was no significant difference between any SF for EPN amplitude.

### (c) Behavioural results

#### (i) Reaction time (RT)

The average RT for correctly identified luminance change trials was *M* = 1.47 s, s.d. = 0.29 s. RTs to correctly identified luminance changes were analysed through a two-way repeated measures ANOVA, to assess the relationship between SF and the emotion of faces presented at the time of the luminance changes on RT. Bonferroni corrections were applied to all *post hoc* tests. A 2 (facial emotion: neutral, fear) × 3 (SF: BSF, HSF, LSF) repeated measures ANOVA revealed a main effect of SF, *F* (2, 650) = 112.36, *p* < 0.001, $\eta_{p}^{2}$ = 0.257, a main effect of emotion, *F* (1, 325) = 89.47, *p* < 0.001, $\eta_{p}^{2}$ = 0.216, but no interaction between SF and emotion, *F* (2, 650) = 2.10, *p* = 0.123. RTs were slower for changes that accompanied fearful faces (*M* = 1.47 s, s.d. = 0.29 s) compared to neutral faces (*M* = 1.46 s, s.d. = 0.30 s). *Post hoc* tests revealed that there were significant differences between all SF levels, BSF and HSF (*t* = 7.74, *p* < 0.001, Cohen’s *d* = 0.08), BSF and LSF (*t* = 14.99, *p* < 0.001, Cohen’s *d* = 0.16) and HSF and LSF (*t* = 7.24, *p* < 0.001, Cohen’s *d* = 0.08). RTs were slowest to BSF (*M* = 1.48 s, s.d. = 0.29 s), followed by HSF (*M* = 1.47 s, s.d. = 0.30 s) and fastest to LSF (1.46 s, s.d. = 0.29 s).

#### (ii) Task accuracy

Hit rates were assessed via the lens of signal detection analysis using the sensitivity measure d prime (*d*’). For each participant, *d*′ was computed by first calculating the hit rate (proportion of luminance change trials correctly identified out of total luminance change trials) and the false alarm rate (proportion of trials incorrectly identified as having luminance changes out of all non-luminance change trials). These were then *z*-transformed (note in instances where hit and false alarm rates were 0 or 1, these were substituted with 1 × 10^−8^ and 0.999999999, respectively, to allow for computation). The difference between these two *z*-transformed statistics was then used to calculate *d*′. The average performance for the luminance change task was *M*(*d*′) = 4.84, s.d. = 2.97, indicating high accuracy overall. A two-way repeated measures ANOVA was run to determine the relationship between SF (BSF, HSF and LSF), the emotion of faces presented with the target (fearful present or neutral) and hit rates as represented by *d*′. The ANOVA revealed no main effect of SF, *F* (2, 70) = 0.01, *p* = 0.989, and no significant interaction between SF and facial emotion, *F* (2, 70) = 7.28, *p* = 0.077; however, the main effect of facial emotion was significant, *F* (1, 35) = 7.03, *p* = 0.012, $\eta_{p}^{2}$ = 0.167, such that *d*′ was greater for neutral faces (*M* = 5.19, s.d. = 3.32) than for fearful faces (*M* = 4.48, s.d. = 2.53). Thus, better performance accompanied neutral compared to fearful faces figure 5.

## 4. Discussion

In this paper, we measured ERP responses to spatially filtered fearful and neutral faces presented to the nasal and temporal hemiretinae in order to determine whether the subcortical pathway relies on LSF content. Overall, we found that the early ERP responses (P1, N170) were faster for neutral BSF and LSF faces compared to neutral HSF faces. There was no interaction between fearful LSF, HSF and BSF faces at P1 and N170, challenging the view that coarse visual information is processed with greater speed via the magnocellular channels [52]. With regard to the hemiretinal manipulation, we uncovered a hemiretinal asymmetry for BSF faces that was not present for HSF faces, as evidenced by a marked increase in neural activity when unfiltered fearful faces were presented to the nasal hemiretina. As the hemiretinal asymmetry is hypothesized to result from differential projections to the superior colliculus, this finding suggests that neural processing in the subcortical pathway is less reliant on HSF information.

### (a) Emotional facial expressions and SF

First, we compared the latency and amplitude of fearful–neutral and neutral–neutral face trials across the three different spatial frequency conditions. We found that the P1 peak latency was sensitive to the filtering manipulation for neutral faces in particular, with HSF faces evoking later peaks than BSF and LSF faces. This is inconsistent with the idea of a rapid processing of the LSF components of a face. Similarly, there was an effect of SF on the N170 peak latency for neutral faces, where both BSF and LSF peaks were earlier than HSF. Thus, our results suggest an interaction between SF and emotional content, where fearful content is processed rapidly regardless of SF, and neutral faces are processed faster at early stages when LSF information is present. Previous literature suggests that LSF content elicits faster visual evoked potential latencies [15,53–55]; however, here we observed this temporal advantage for neutral faces only. This latency advantage for neutral LSF faces only appeared over early epochs, as the EPN latencies for both filtered conditions were equivalent. The typical early processing effect on latency has been attributed to the speed of the neural channels carrying LSF information [52,56–61]. LSF information is encoded preferentially through magnocellular and HSF through parvocellular connections [1]. The speed at which magnocellular and parvocellular input reaches the primary visual cortex is dissociable, with magnocellular input arriving 20–25 ms faster than parvocellular input [52,62]. Yet this does not align with our results, whereby we would expect magnocellular input for all faces (fearful and neutral) to show this latency effect. Several explanations exist for this inconsistency. For example, it is possible that all fearful faces, regardless of SF content, are encoded at the same rate owing to their emotional content. Since the neutral faces are devoid of this preferentially processed emotional content, this may allow for the BSF and LSF latency advantage to appear. In this way, our results support an interaction between SF and emotional content.

Regarding amplitude, the earliest effects of SF and emotion were found at N170. We found a reduced N170 in response to all filtered faces. This is in line with research showing that any filtering suppresses N170 peaks [63]. Filtering might also explain why we found no enhancement of the N170 for fearful faces; while the N170 is typically enhanced for emotional expressions [64], this enhancement is suppressed by spatial frequency filters [63,65]. Previous monocular hemiretinal viewing paradigms have also found no emotion-based N170 difference [32]. This could be attributed to the diffusion or diversion of attention. For example, the effects of emotion on lateralized stimuli have been reportedly suppressed by diverting attention to a central task [66] and studies where emotional faces are presented in the periphery have suggested that allocation of attention is required for N170 modulation by emotion [67–69]. Therefore, as the present experiment required the participants to direct their attention towards the centre of the screen, it is likely that attention towards the fearful faces was insufficient to enhance the N170.

EPN analysis revealed interactive modulations based on SF and emotion conditions. Typically, greater negative EPN deflections are linked to negative affective content and greater attentional deployment [65–70]. In keeping with this, we found that fearful faces evoked greater EPN deflections than neutral faces, and that LSF faces evoked greater EPN deflections than BSF and HSF faces. These findings suggest that fearful facial expressions and LSF faces require greater attentional resources. Discrete pairwise comparisons revealed that the LSF faces (i.e. fearful and neutral) evoked greater negative deflections than all other conditions aside from HSF fearful faces [65]. This could indicate that greater attention was allocated to LSF faces, regardless of their facial expression, potentially thanks to rapid magnocellular processing enabling increased attentional allocation.

The behavioural results affirm the validity of our paradigm and methodology; the manipulation of SF did not change task accuracy, indicating that performance was equivalent across the three blocks and that participants effectively fixated the centre of the screen on the central task. However, SF did impact RT, with the slowest responses to BSF compared to both filtered conditions and fastest to LSF. This suggests that intact, unfiltered images provide the greatest task interference through attentional distraction. We also found differences in accuracy and RT dependent on the facial expressions present in each trial. Accuracy was higher, and RTs were faster for trials that featured neutral–neutral face pairs compared to fearful–neutral face pairs, consistent with the well-established attentional engagement associated with emotional content [51,70].

This overall pattern of results supports an interaction between SF and emotional content with regards to temporal dynamics and cognitive processes. Crucially, these findings cannot be attributed to differences in contrast across SF conditions because contrast was corrected in the current procedure [20]. However, these results alone do not conclusively support the idea of rapid processing occurring through subcortical projections.

### (b) Hemiretinal processing

Our findings demonstrate a hemiretinal asymmetry for unattended emotional faces (as in [32,34]), which is not apparent for HSF visual information. A significant difference was found in the hemispheric difference waveform between temporal and nasal presentations; a clear hemiretinal asymmetry was present for BSF faces but not HSF faces (see figure 4*b*). Follow-up tests revealed that this effect was driven by differences when stimuli were presented to the nasal hemiretina. The nasal presentation of BSF faces evoked a negative N170 difference waveform deflection, which was not evoked by the nasal presentation of HSF faces (see figure 4*a*). As projections from the nasal hemiretina send a greater proportion of information to the superior colliculus [23], these results can be interpreted as a decreased sensitivity of the nasal hemiretina (and the subcortical pathway) to HSFs.

We aimed to assess whether the subcortical pathway is sensitive to LSF information by taking advantage of functional and anatomical hemiretinal asymmetry [23–25,71–74]. The BSF waveform shows the neural product of the hemiretinal projection asymmetry. In comparison, the SF asymmetries indicate the visual information to which these pathways are sensitive. The HSF waveform differs significantly from the BSF, while the LSF does not differ from either. The follow-up comparisons show that HSF faces differ from BSF processing owing to differences in nasal hemiretinal processing. A close inspection of figure 4*a* reveals a strong negative deflection in the nasal BSF condition that occurs at the time of the N170, but this deflection does not occur in the HSF condition. While the LSF waveform is not significantly different from the BSF, visual appraisal reveals a much weaker deflection. The implications of this pattern of results are discussed further in subsequent sections.

Our conclusions are constrained by several methodological limitations. First, while the bilateral presentation of faces was necessary to explore the N2pc, this limited investigation of the hemiretinal asymmetry to emotional faces. Future research could expand the stimulus selection to include scrambled or inverted faces, which would allow for an assessment of intact, neutral faces. Another issue pertains to the specific SF parameters that were employed. While the SF ranges were selected based on prior research, it remains possible that the subcortical pathway is sensitive to SF ranges outside of those included in this study. Development of methods to measure sensitivity to SF content without making *a priori* assumptions about how to delineate SF conditions will be vital to advance our theories of how the subcortex contributes to primate vision.

#### (i) Effect of filtering

Our findings provide evidence that the subcortical pathway is insensitive to HSF information presented in isolation [75–78]. This makes sense in the light of other findings showing that LSF is crucial for emotion perception and is related to subcortical activation [8,79–81], while HSF information is essential to later discriminatory processes like facial familiarity recognition and is related to fusiform cortex activation [8,82]. However, the current results do not rule out the use of HSF information in emotion processing or early processing but rather indicate that HSF information is less involved in rapid subcortical processing specifically. Subcortical activation modulates and predicts the activation of higher cortical areas [83–86], indicating that early subcortical processing guides attention as directed by LSF information followed by key HSF contributions.

The hemiretinal difference waveform evoked by LSF fearful faces was not significantly different from BSF or HSF fearful faces. This suggests that the subcortical pathway is not solely activated by LSF content in visual signals. This appears to diverge from the literature, indicating a reliance on LSF by the subcortical pathway. For example, intracranial recordings in the human brain show that amygdala neurons respond very quickly (within ~72 ms) to LSF input of fearful faces, supporting this idea of subcortical pathway function [8,87–89]. Evidence from patients with V1 damage and blindsight has shown that LSF fearful faces enhance amygdala activation relative to HSF stimuli, likely through subcortical connections [7]. In healthy individuals, event-related fMRI paradigms have also shown that, in addition to increased amygdala activity, LSF fearful faces activate other neural regions thought to form part of the subcortical pathway to a greater extent than HSF faces [8]. However, other research aligns with our finding that the subcortical pathway may not be specifically tuned to LSF information [20].

Multiple diverging conclusions can be drawn from this pattern of results. First, it is possible that despite our efforts to select the range of LSFs to whichthe amygdala is sensitive, the subcortical pathway may be differentially sensitive to a frequency range that we did not employ here. For this interpretation to be ruled out, research must employ stimuli filtered to a range of SF information, as in [90,91]. It is also possible that the subcortical pathway receives a combination of SF input and is not preferentially tuned to either low or high SF information (aligning with the findings of McFadyen *et al*. [20]). This is the most compelling interpretation of the results, coupled with the notion that while the subcortical pathway is not dependent on LSF information, it has a comparative bias against HSF information at the frequency range presented.

Research has linked subcortical processing to faces presented below awareness. For example, one study with intracellular recordings in the insula and amygdala found earlier responses to low SF emotional faces compared to high SF emotional faces when participants were aware of faces. However, when participants were unaware of faces, they found early responses across a range of SFs [90], a finding that has been supplemented by behavioural research [92]. However, this approach is yet to be combined with hemiretinal methodology.

## 5. Conclusion

In summary, our results showed that neutral expressions were processed faster by the brain when comprising BSF and LSF than HSF information, but fearful faces showed no such effect, suggesting that magnocellular input interacts with emotional content to impact processing speed. Importantly, we found the predicted hemiretinal asymmetry for faces in the BSF condition, but not faces in the HSF condition. On the other hand, the LSF condition did not differ from either. As the nasal hemiretina is thought to project more extensively to the superior colliculus, the findings suggest that subcortical processing relies on information delivered in a combination of frequencies, but with a decreased sensitivity for HSF information.

## Data Availability

Data and Code can be accessed through the Open Science Framework [93].
